# The experience of live-remote exercise—perspectives after cancer treatment

**DOI:** 10.1007/s00520-024-08736-4

**Published:** 2024-07-18

**Authors:** Melissa Kotte, Cecilia H. Ringborg, Yvonne Wengström

**Affiliations:** 1 Department of Neurobiology, Care Sciences and Society, Karolinska Institutet, Alfred Nobels Allé 23, 141 83 Huddinge, Stockholm, Sweden; 2 Medical Unit Breast, Theme Cancer, Endocrine Tumors and Sarcoma, Karolinska University Hospital, Stockholm, Sweden

**Keywords:** Online exercise, Virtual supervision, Physical activity, Oncology, Experience, Qualitative study

## Abstract

**Purpose:**

Live-remote exercise interventions, supervised by exercise professionals in a home-based setting, could potentially enhance exercise accessibility for cancer survivors, yet research on their perspectives is limited. This study explored cancer survivors’ experience of exercise within the context of a live-remote exercise intervention, to understand factors influencing exercise engagement.

**Methods:**

Four online focus groups with, in total, 22 breast, prostate, and colorectal cancer survivors were conducted between March and June 2023. These individuals had participated in a 12-week live-remote exercise intervention. The semi-structured discussions were transcribed verbatim and analysed using reflexive thematic analysis with an abductive approach. The Capability, Opportunity, Motivation model of Behaviour (COM-B) served as a supportive framework.

**Results:**

Nine themes were identified and mapped onto COM-B domains (capability, opportunity, motivation). Factors influencing cancer survivors’ exercise engagement included exercise readiness following cancer treatment, bringing exercise closer, in capable hands, peer support through shared experience, life factors as hurdles or support, exercise as an integral component of cancer treatment, caring for myself and others after me, the positive impact of exercise exceeding expectations, and getting into the habit.

**Conclusions:**

Identifying factors shaping exercise engagement, these findings emphasise live-remote’s potential benefit in overcoming barriers and fostering participation. Supervised by professionals, it offered psychosocial and exercise support, facilitating the integration of exercise into daily life.

**Implications for cancer survivors:**

Elucidating key factors for exercise engagement within a live-remote context is essential for developing and implementing live-remote exercise interventions to ensure accessible, integrated exercise for optimal post-treatment well-being for cancer survivors.

**Supplementary Information:**

The online version contains supplementary material available at 10.1007/s00520-024-08736-4.

## Introduction

Regular exercise has been shown to have beneficial health effects in cancer survivors, including improving health-related quality of life (HRQoL) and other important health and survival outcomes [1–5]. Moreover, supervised in-person exercise programmes have been proven to be more effective, as well as safe and feasible [1, 2, 6]. Supervised exercise is therefore recommended as a standard of care for individuals treated for cancer with international guidelines existing since 2010 [7]. Despite this, the majority of cancer survivors fail to meet these guidelines [8, 9] due to a number of identified barriers. Not only is there limited access to onsite exercise facilities with specialised exercise support, but cancer survivors also report barriers to in-person attendance, including travel distance and time constraints [10–12].

Providing easily accessible, high-quality exercise support and programmes to cancer survivors thus poses a significant challenge. An innovative solution to improve the availability and accessibility involves employing videoconferencing platforms such as Zoom, Skype, Microsoft Teams, or FaceTime, for live-remote exercise delivery. This format involves real-time online group exercise sessions conducted remotely, with virtual interaction and supervision by an exercise specialist in a home-based setting. The COVID-19 pandemic prompted a shift from in-person exercise classes to live-remote formats, leading to research into the viability and efficacy of this mode of delivery [13]. However, research on live-remote exercise for cancer survivors remains limited, with a lack of adequately designed larger randomised controlled trials [13].

EX-MED Cancer (exercise medicine for people with cancer) Sweden is a novel and innovative live-remote exercise intervention delivered using the videoconferencing platform Microsoft Teams. Participants engage in two 60-min exercise sessions per week for 12 weeks, incorporating both resistance and aerobic exercises, conducted by personal trainers in groups of up to eight. These trainers, from community gyms, have completed specialised exercise oncology education modules. Before starting, participants receive an in-person orientation, exercise materials, and solutions for any identified barriers. Each session includes a warm-up, progressive circuit training, and cool-down, with intensity tailored to individual responses and monitored by the trainer to ensure safety and proper technique. This live-remote exercise intervention is being tested in a randomised controlled trial in people who have been treated for breast, prostate, or colorectal cancer. Details of the trial protocol can be found elsewhere [14].

The EX-MED Cancer Sweden intervention design incorporates strategies to address known barriers to exercise participation and leverages commonly identified facilitators of exercise. However, it is important to note that existing research on barriers and facilitators to exercise among cancer survivors has predominantly focused on the period preceding the introduction of a live-remote exercise delivery approach [10–12]. Limited research exists that explores the perspectives of cancer survivors on exercise participation within the context of live-remote exercise.

This qualitative study aimed to explore cancer survivors’ experience of exercise within the context of a live-remote exercise intervention. A qualitative approach was chosen to gain an understanding of the factors influencing exercise engagement in individuals who have completed cancer treatment, with the goal of informing the design and implementation of future live-remote exercise programmes and enhancing exercise participation among cancer survivors.

## Theoretical framework

This study applies the *Capability*, *Opportunity*, *Motivation model of Behaviour* (COM-B) [15], as a supportive framework. The COM-B model serves as a tool to support determining barriers and facilitators to a positive health behaviour, in this case, exercise. This approach enables the tailoring of interventions that target the behaviour. The model identifies the interlinked elements of capability (physical, psychological), opportunity (physical, social), and motivation (reflective, automatic) that influence the health behaviour [15].

The authors chose the COM-B model because it provides a useful framework for supporting the abductive reasoning within the analysis to identify factors that influence cancer survivors’ participation in exercise. By defining these factors, it becomes possible to further develop and refine interventions designed to promote exercise participation among cancer survivors.

## Methods

### Design

A qualitative explorative study using focus groups was conducted to gain a broad insight into the participant’s experiences. Data was analysed thematically using an abductive approach to deepen the understanding of the factors influencing the participant’s engagement in live-remote exercise. This study followed the Standards for Reporting Qualitative Research (SRQR) guidelines for qualitative studies [16].

### Participants

This focus group study was conducted between 03/2023 and 06/2023 as part of a larger randomised control trial (the EX-MED Cancer Sweden trial) investigating a live-remote exercise intervention for people treated for breast, prostate, or colorectal cancer in Sweden [14]. Participants were eligible for the EX-MED Cancer Sweden trial if they (1) were ≥ 18 years of age; (2) were diagnosed with any type of stage I–IIIa breast, prostate, or colorectal cancer; (3) had undergone and completed curative treatment within the last 5 years (eligible if currently receiving/scheduled to receive anti-hormonal therapy); and (4) were able to read and speak Swedish. Participants were eligible for the present qualitative study if they, in addition, had participated in the 12-week live-remote exercise intervention of the EX-MED Cancer Sweden trial. A total of 43 eligible participants were invited to participate in the focus groups, of which 22 were accepted.

### Data collection

To explore participants’ experiences, we used a semi-structured approach. An interview guide was developed by three researchers (MK, CR, YW), inspired by the COM-B model [15], and contained broad and in-depth, open-ended questions with topics focusing on the experience of exercise after cancer treatment and factors that influenced exercise participation in the context of participating in a live-remote exercise intervention (see Supplement 1). The interview guide was piloted in the first focus group with no changes being made for the remaining focus groups. Probing questions were used to ensure richness and complexity of the data collected [17]. Discussions were not limited to the topic areas, providing the opportunity for the exploration of additional topics related to exercise participation brought up by the focus group participants.

Four focus groups were conducted with five to six participants in each. Participants were allocated to a focus group based on their convenience while also ensuring a mix of participants from the different exercise intervention groups. The adequacy of the data to answer the research question guided the number of focus groups conducted [17]. The focus groups were moderated by one researcher. A second researcher assisted by asking additional probing questions when needed and summarising key points. Each focus group lasted between 50 and 70 min and was conducted online via the video-conferencing platform Zoom. This online method was chosen as it is a method considered most similar to face-to-face, is convenient for participants [18], and the exercise intervention had also been delivered online via video-conferencing (Microsoft Teams). The group discussions were audio- and video-recorded and transcribed verbatim by a professional transcriber. Sociodemographic characteristics and medical information were collected as part of the larger EX-MED Cancer Sweden trial.

### Data analysis

Data from the focus groups were analysed using reflexive thematic analysis with a contextualist approach [19, 20]. Acknowledging that the knowledge produced is local, situational, and provisional, we sought to provide a rich, contextualised examination of the experience of exercise participation in cancer survivors to contribute to our existing understanding of this topic. Through the analysis, we sought to identify patterns across the data in order to convey the experiences of the cancer survivors participating in the exercise.

The analysis was abductive, shifting between an inductive approach to identify the themes and then moving to a deductive approach as the themes were mapped onto the COM-B model [15]. The inductive stage of the analysis was grounded in the data in a “bottom-up” approach in which there was no attempt to fit it into the existing model. In accordance with Braun and Clarke [19, 20], this process included familiarisation with collected data, involving repeated listening to the recorded data and reading and re-reading the transcribed data. Relevant sections of data were collated and coded, and candidate themes were generated based on shared patterned meaning across the dataset which were then reviewed and refined, with final themes named and defined. Themes were identified at a semantic level, reflecting the concepts directly discussed by the participants.

All authors conducted the data analysis. Coding and theme construction were consultive and iterative, as emergent codes and themes were discussed and refined, ensuring a reflexive and rigorous process was applied to the analysis [19, 21]. The process of data collection and analysis was undertaken through the lens of our background as registered nurses in cancer care and exercise research and beliefs in the value of exercise. Two of the authors (MK, YW) are involved in the randomised controlled trial. We reflected over our subjectivity by engaging in regular discussions and maintaining an analysis journal [19, 21]. Data excerpts chosen to illustrate each theme [21] have been translated into English and edited to facilitate readability, with the emphasis reflecting the participant’s original expression. In the final stage of the analysis, a deductive approach was applied to map the generated themes onto the COM-B model [15].

*Ethical considerations*

Ethical approval for this study was granted by the Swedish Ethical Review Authority (2019–04,151, 2021–01,715). All participants provided written informed consent prior to data collection.

## Results

### Participant characteristics

Participants in this study were aged from 45 to 78 years, with an average age of 59.5 years (SD = 10.4). The majority of the participants were female (16/22), 15 had been treated for breast cancer, four had been treated for prostate cancer, and three had been treated for colorectal cancer. Time since exercise intervention completion ranged from 3 to 48 weeks, with an average time of 19 weeks (SD = 13.3). Participant characteristics are described in Table 1.

**Table 1:** Focus group participant characteristics

Characteristic	*n* (%)
Sex	
Male	6 (27.3)
Female	16 (72.7)
Age (years)	
45–54	9 (40.9)
55–64	5 (22.7)
65–74	6 (27.3)
> 75	2 (9.1)
Average age Years (standard deviation)	59.5 (10.4)
Cancer diagnosis	
Breast	15 (68.2)
Prostate	4 (18.2)
Colorectal	3 (13.6)
Time since intervention completion (weeks)	
1–9	5 (22.7)
10–19	10 (45.5)
20–29	1 (4.5)
30–39	4 (18.2)
> 40	2 (9.1)
Average time since intervention completion Weeks (standard deviation)	19 (13.3)

### Experiences of exercise after treatment for cancer

The analysis process resulted in nine key themes: (1) exercise readiness following cancer treatment, (2) bringing exercise closer, (3) in capable hands, (4) peer support through shared experience, (5) life factors as hurdles or support, (6) exercise as an integral component of cancer treatment, (7) caring for myself and others after me, (8) the positive impact of exercise exceeding expectations, and (9) getting into the habit.

1. **Exercise readiness following cancer treatment**

All participants in the focus groups had some form of prior exercise experience, although for some, it had been quite some time since they had engaged in regular exercise. Some had been regular exercisers while others had exercised periodically. Several participants expressed that their past experience with exercise had been a positive aspect of their lives and that they had aspired to return to it, viewing it as a preferable alternative to medication.

*I used to exercise before I got sick, and then I felt that it was something positive. It’s always better, I think, to exercise than to take medicine.* (Female, 51, breast cancer)

Participants shared a general awareness of the importance of exercise and physical activity, even after a cancer diagnosis. However, they all reported a lack of specific knowledge regarding exercising after cancer treatment. Some described receiving general advice from healthcare professionals who emphasised the benefits of staying active, yet they expressed a strong desire for concrete guidance on exercise recommendations to engage in, how much they could push themselves, and what would be appropriate for their situation. Some participants mentioned receiving conflicting messages and being told to stay active but not to overexert themselves. One participant described a conversation together with a healthcare professional:

*“It’s good if you are physically active”, that’s all that was said. So I tried to ask more, “So, how physically active should I be, should I walk ten thousand steps a day?” The response was, “No, that’s far too much. If you are just a little bit active, you’ll be fine. If you’re taking walks, then that’s enough”. *(Female, 47, breast cancer)

Despite not receiving specific advice, the participants generally expressed a lack of concern about engaging in exercise after cancer treatment. In fact, before the start of the EX-MED Cancer exercise intervention, they eagerly anticipated it. However, several participants reported feeling uncertain about their own ability to exercise due to the physical toll of treatment, describing needing a push to dare to really exert themselves.

*It was like you needed a little push to even dare to really try, really dare to go for it, because it’s one thing to go out for a fast walk, but it’s another thing to push yourself almost to the max.* (Female, 45, breast cancer)

The first exercise session was really challenging for some, and they questioned whether they would make it through the 12-week programme. Nonetheless, they had confidence in the expertise of the programme facilitators and were even surprised at their own ability to perform the exercises, which they felt improved with each session.

2) **Bringing exercise closer**

Interestingly, the participants did not encounter significant difficulties when using the videoconferencing platform Teams for exercising. Although a few mentioned occasional technical hiccups, they found these issues easily resolvable and not disruptive to the exercise sessions. They attributed this to the prevalence of videoconferencing technology usage in the wake of the COVID-19 pandemic, which made them more accustomed to its use in various settings. The participants expressed gratitude for the virtual format, as it removed some of the barriers that had previously deterred them from going to an on-site facility. However, some scepticism was expressed before the start of the intervention regarding exercising “in front of a screen” and there were doubts about the intensity of the workout. Nonetheless, participants were surprised to discover that they experienced an effective workout.

The live-remote exercise offered the participants greater accessibility compared to traditional on-site workouts. Convenience was a key factor described by all the participants and for some the live-remote format was a prerequisite for participation. Live-remote sessions eliminated the hassle of getting to a physical gym. It allowed individuals to integrate exercise into their daily routines, whether at home, work, or while on vacation. Time-saving and efficient, it helped participants find a place for exercise within their lives.

*It was great that it was digital as it only took an hour. I mean if you have to travel somewhere with the car or bus it’s an hour there and an hour home. You also weren’t tied to your home. You could train other places too, which I appreciated.* (Male, 63, prostate cancer)

Participants appreciated the virtual format because it allowed them to exercise when they did not feel up to visiting a gym. They described feeling self-conscious about their appearance due to hair loss, scars, or missing a breast, as well as concerns about open wounds and susceptibility to infections.

*I had problems with a wound that wouldn’t heal so that’s also what has been nice about training digitally. If I was to go to a gym with a weeping wound, that wouldn’t have felt so great. But to be able to be at home in my own environment and exercise has worked out really well. *(Female, 51, breast cancer)

In a gym setting, they expressed feeling like outsiders and that live-remote offered an experience that was unpretentious and less competitive. The participants appreciated that it did not matter whether they had just come from the breakfast table or what they were wearing; they could concentrate on their training instead of worrying about appearances. Interestingly, the participants did not consider it overly intimate, even though they had glimpses into each other’s homes and vice versa.

3) **In capable hands**

The personal trainer assumed a central role for participants, becoming not only an exercise instructor but also a source of inspiration and motivation, pushing them to exceed their perceived limits. Some of the participants described initial doubts about connecting with the personal trainer virtually; however, as the sessions unfolded, these doubts dissipated, replaced by a genuine bond between the personal trainer and participants.

*I felt that the personal trainer could really see us and had the ability to engage us, despite the physical distance that arises when she’s on the screen and we’re in our respective rooms.* (Female, 47, breast cancer)

The participants valued the personal trainer’s ability to tailor exercises to their unique needs. It was not just about following generic instructions; it was personalised guidance despite being in a virtual group setting. Whether someone was dealing with pain or stiffness, or needed help with technique, the personal trainer offered individualised instruction that provided a sense of safety and reassurance.

*It was great to have a personal trainer who could actually delve deep and see, and you were able to ask all of these questions that you had. “How should I do this? My arm feels stiff, what should I do?” And she helped a lot, with pain you still have in places, from surgery and other things. You got excellent tips directly from her.* (Female, 70, breast cancer)

For all participants, the personal trainer played an instrumental role in their engagement and confidence in the live-remote exercise intervention, and it was expressed that the personal trainer was the keystone of the success of the entire intervention.

4) **Peer support through shared experienced**

Participants expressed a feeling of peer support in their collective exercise experience, describing it as something social. Some even reported connections being fostered beyond the group sessions. At the outset, participants felt that it was fairly anonymous, but as time passed, the groups grew closer. Any scepticism to training in a group was quickly overcome as the sense of togetherness became of value. Familiarity with one another’s voices and the encouraging sounds of exertion became comforting.

The significance of exercising with those who had endured similar challenges, such as difficult treatments and hair loss, was a common sentiment. This shared background created a safe environment where the participants felt less vulnerable due to physical limitations and believed there was a deeper understanding among peers.

*To be able to do it this way, in a group where there are others who have had similar experiences. You may have gone through a pretty tough treatment. You may have had surgery. You know a little bit where everyone is coming from. That made it so much easier.* (Female, 52, breast cancer)

Interestingly, one participant viewed their fellow participants as training companions rather than individuals who had also had cancer, experiencing the same sense of familiarity felt when participating in group training prior to being diagnosed with cancer.

5) **Life factors as hurdles or support**

Most of the participants described various factors that represented hurdles to them incorporating exercise into their daily lives. Obtaining a gym membership was easy; however, actually getting to the gym was difficult. The most common factors described were lack of time due to work and family commitments, and the burden of travel. Participants who had returned to full-time work reported difficulty in finding time to exercise. Several described that once they had returned to work post-treatment there was an expectation that they should function at full capacity, leaving little room for exercise. They felt that both employers and social security (who fund sick leave) overlooked the physical toll caused by treatment.

*I didn’t have the energy to keep arguing with them [social security] anymore about me being on sick leave. They thought that because I had finished treatment, I was healthy. But they don’t see everything you have gone through and how much of a beating your body has actually taken.* (Female, 47, breast cancer)

Commuting to an onsite facility also proved for many to be a logistical challenge. One participant talked about crowded classes at the gym and instructors being unable to provide personalised attention adding to the hurdles. Some participants described barriers that extended to concerns about how others perceived and even reacted to their appearance when they exercised.

*I decided to go swimming, and I went to the pool once it [mastectomy wound] had healed. Then I get totally told off by this woman there. She said, “How can you show yourself like this in public? How can you scare people like this?” So nothing became of that [swimming] either. I really became discouraged from going anywhere.* (Female, 52, breast cancer)

The participants who worked full-time and participated in the exercise intervention described being met by varying degrees of support from their employers. Some enjoyed the backing of supportive employers who encouraged their participation. In contrast, others faced resistance if they attempted to exercise during working hours, even if they diligently made up the time.

However, not all the participants experienced hurdles. Some participants shared stories of support and encouragement from their immediate surroundings. Those who were no longer working full-time, such as retirees or semi-retired individuals, reported fewer external barriers to exercising. Even those who found the logistics of family life challenging described being supported by their family members in participating in the exercise intervention. In certain households, exercising in front of the computer became the norm, with family members respecting the designated exercise times.

6) **Exercise as an integral component of cancer treatment**

The participants believed that tailored exercise programmes should be offered to all individuals following cancer treatment. Moreover, they felt that exercise programmes should be an integral part of the treatment package. It was likened to the routine nature of mammography screening in post-treatment follow-up. Some suggested that it should already be offered during treatment.

An emphasis on the medical aspects of treatment was expressed and once treatment was completed, participants reported feeling a sense that something was missing, that they had not received the support needed to fully transition back to regular life. Attending the clinical rehabilitation post-treatment which included some aspects of physical activity was highly valued; however, most of the participants had not been provided with this opportunity.

The consensus was that exercise should be seen as a transition step toward returning to a sense of normalcy and that exercise programmes should be accessible to everyone without the need to ask or pester for it. They believed the healthcare system should take a more proactive approach to offer exercise programmes as part of their services, given the positive effects they can yield, not just for themselves but also for employers and society.

It would be good if it was a part of the established treatment program, so that it’s not just up to yourself. It’s hard to know when you’ve only been through something like this once yourself, but the healthcare service has been through these situations over and over again and know what works. Therefore, taking the initiative on an individual level is difficult, to decide, yes, I’m going to go to the gym to get better, so to speak. (Female, 68, breast cancer)

7) **Caring for myself and others after me**

Participants emphasised the significance of self-care after the toll that treatment took on their bodies, expressing a strong desire to regain their former selves. They viewed exercise as a form of self-care to improve health and well-being, manage side effects, and help to return to a sense of normalcy. Furthermore, they emphasised the importance of finding a solution that meant not having to take medication.

*The treatment is tough, with all the medication, and it takes a toll on you in every possible way. Your body goes through so much, and then you reach a point where you think, I’ll do whatever it takes. I just want to feel like my old self again.* (Female, 57, breast cancer)

Moreover, they were motivated not only by their own well-being but also by altruism. They expressed a desire to contribute to research that could help those diagnosed with cancer in the future. Another aspect mentioned was exercising to maintain good health to be able to take care of family and children.

*We had our second child just when I started my treatment, and it’s made me realise how important training is. Because I need to try to keep myself alive, for the sake of my children. I have very young children and I want to be there for them when they grow up. *(Male, 49, colorectal cancer)

8) **The positive impact of exercise exceeding expectations**

Participants expressed a sense of anticipation about the exercise programme, expecting to benefit from it. However, their experiences went beyond their initial expectations. Some described being positively surprised by the effects after just one or two sessions. The programme design was highly valued for the array of benefits experienced. For instance, improved strength and balance, as well as increased stamina and fitness levels, allow them to engage in daily activities more easily, like walking uphill or climbing stairs without support.

*I was hoping to feel better, and it became noticeable quite quickly, in such simple everyday tasks like not having to hold the handrail when climbing the stairs. And then you feel you get a push, that it just gets better and better. *(Male, 76, colorectal cancer)

Furthermore, they reported experiencing less pain, reduced swelling, better sleep, increased energy levels, and reduced fatigue. Some participants who reported suffering from muscle and joint pain due to anti-hormonal treatment found relief through exercise.

*I felt so much better. It was like an awakening for me, that I became more energetic and the pain that I had on the side where I received radiation, it disappeared. And the mobility in my arm returned.* (Female, 65, breast cancer)

The positive effects extended to their mental well-being, making them feel mentally stronger, happier, and more positive; the exercise intervention contributed to recovery from mental illness. Although all the participants described positive effects, some described their battle with “chemo-brain,” experiencing fatigue and memory loss. They felt unsure how much exercise had improved these side-effects, however believed that they might have felt worse without training. Some of the participants who had not continued their training beyond the exercise intervention reported noticing a decline in their physical and mental well-being.

In general, they described a feeling of their bodies recovering more quickly from their cancer treatment which in turn boosted their motivation to exercise. The positive effects served as strong motivators to partake in the exercise sessions and for some to continue to exercise beyond the intervention. While some participants found it challenging to get going on certain days, they found that the post-workout feeling of well-being motivated them for the next session.

9) **Getting into the habit**

Some of the participants, who described themselves as struggling with laziness and procrastination in establishing exercise routines, found themselves feeling a sense of obligation to show up. The scheduled sessions were highly valued. They recognised that without this regular commitment, exercise might not have become a part of their weekly routine.

*We had training Mondays and Thursdays at 18:00 which suited me really well. When I first joined the study though I thought I could just connect online and train whenever I wanted, so when I found out it was scheduled times, I was a bit like, “What, is this a fixed time? How is this going to go? I don’t have time for that”. But I understand why in retrospect. I would never have done it otherwise. *(Female, 53, colorectal cancer)

Not everyone maintained their exercise routines beyond the intervention. Several participants reported no longer exercising or only engaging in sporadic physical activity. To some of them, this was not a surprise, as they recognised their own tendencies. Maintaining their commitment without the supportive framework of the intervention proved challenging. They acknowledged that during the intervention, exercise had become a habitual part of their routine, but once it concluded, they regressed to their previous habits.

However, for some, participating in the exercise intervention became a gateway to establishing lasting exercise routines. They continued with the programme after its conclusion, while others ventured into new forms of exercise, or returned to previous exercise habits they had abandoned.

### Themes in relation to the COM-B model

**Fig. 1:**
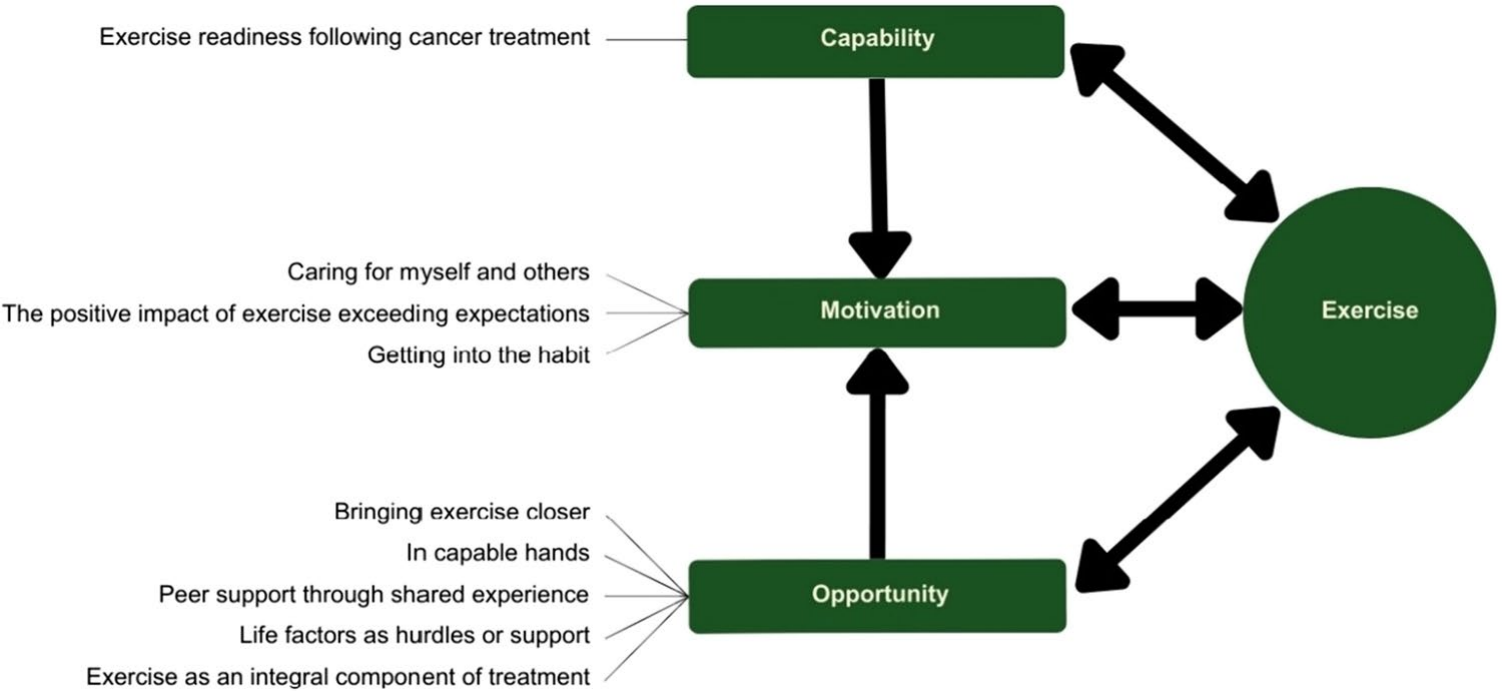
Themes mapped onto the COM-B model

The nine themes were mapped onto the COM-B model (Fig. 1): one theme related to capability, five themes related to opportunity, and three themes related to motivation.

## Discussion

This qualitative study offers novel insights into the post-cancer treatment exercise experience within the context of a home-based live-remote exercise intervention. The barriers hindering in-person exercise attendance, as outlined by participants, encompassed factors such as travel distance and time constraints due to work or family commitments. These findings resonate with prior research delineating barriers to exercise engagement among individuals undergoing or having completed cancer treatment [10, 11, 22]. The live-remote format, however, proved instrumental in overcoming these barriers, therefore providing the participants the opportunity to integrate exercise into daily routines. Furthermore, this format allowed participants to overcome concerns about physical appearance, health risks, and the competitive atmosphere that some associated with public exercise settings. Older people participating in a recent live-remote yoga trial reported finding the live-remote format superior to an in-person format for similar reasons [23].

The live-remote format also allowed for supervision by an exercise professional, something that has long been recognised as a key facilitator for exercise, with studies consistently demonstrating the superiority of supervised exercise programmes in terms of effectiveness and adherence [1, 2, 24]. Therefore, it comes as no surprise that the role of the personal trainer in the present study was deemed crucial by the participants. What was unexpected, however, was the participants’ genuine sense of connection and support from the personal trainer, despite the live-remote format. Additionally, participants reported experiencing a supportive atmosphere among their fellow exercisers. Exercising in-person in a group with other cancer survivors has previously been shown to provide valuable psychosocial support, which can consequently enhance exercise engagement and attendance [11, 25]. Simply sharing a cancer experience may, however, not suffice to foster meaningful relationships that promote sustained attendance and engagement [26, 27], particularly in an online setting where opportunities for social interaction may be limited [28]. A key element contributing to the participants’ sense of support in the present study may stem from their continual participation in the same exercise group for the 12-week intervention period. This continuity contrasts with exercise classes where members frequently join and leave, which hinders the formation of meaningful connections and a cohesive group dynamic [27].

Furthermore, exercise professionals in a live-remote context shoulder a greater responsibility to facilitate a supportive environment [27, 28]. Craig et al. [27] provide a comprehensive array of supportive behaviours that exercise professionals can employ to facilitate social support online, including encouraging socialisation before and after classes, promoting connections outside of classes, making themselves available as a support person, providing resources and advice related to cancer, and discussing and empathising with the cancer experience. However, it is important to acknowledge that exercise professionals themselves require support and training to effectively foster a supportive online environment [27]. Therefore, further qualitative research would benefit from exploring the needs of exercise professionals delivering online classes to cancer survivors and developing strategies and training programmes to meet those needs.

While participants in the present study did not express any concerns regarding privacy or the presence of other household members during the home-based sessions, it is crucial to acknowledge these potential limitations and consideration must be given to these ethical issues when delivering sessions in a live-remote format [28, 29]. Privacy concerns, for instance, may result in a reluctance to activate the camera during sessions [28], potentially hampering social interaction and supervision. Conversely, involving other household members, such as children or spouses, may present an opportunity for additional social interaction and support [28]. These dynamics underscore the importance of carefully navigating privacy concerns and leveraging the potential benefits of involving household members in live-remote exercise programmes.

Interestingly, participants in the present study did not encounter barriers to utilising the online videoconferencing platform, in contrast to recent exercise trials utilising a live-remote format during the COVID-19 pandemic [23, 30]. Strategies were implemented to facilitate virtual participation in our study, including instructions on using the video-conferencing platform and a researcher present for the first online session to troubleshoot any technical issues, which may partially account for these findings. However, it is plausible that individuals with limited digital literacy opted out of participation. Despite the increasing prevalence of digital health services, including supportive care services for individuals with cancer [31, 32], challenges associated with digital literacy have been reported as a barrier in the context of live-remote exercise programmes [30, 33]. Notably, older adults, in particular, may lack the necessary proficiency to effectively navigate digital platforms to support exercise endeavours [34]. This underscores a critical ethical consideration in the development of live-remote exercise interventions [29]. Consequently, digital support strategies must be integrated into the design of live-remote exercise interventions to ensure their successful implementation and adoption, including allocating dedicated time and resources to ensure digital competency and adoption among participants [30].

Despite expressing motivation to engage in exercise for health benefits and post-cancer treatment recovery, participants in the present study also voiced concerns about their capability. These concerns were related to their physical ability as a result of cancer treatment and their knowledge of appropriate exercise post-treatment. Hesitancy to exercise due to lack of knowledge about exercising following cancer treatment is a well-documented issue [11, 35]. While healthcare professionals play a crucial role in providing physical activity advice and support for cancer survivors, the participants in the present study reported lacking this and, at times, received conflicting information. Findings from past studies have also suggested that inadequate information and support from healthcare professionals are barriers for cancer survivors engaging in physical activity and exercise [10, 12, 35].

Although healthcare professionals recognise the importance of discussing physical activity, many lack the necessary expertise to offer specific guidance and encounter barriers in referring cancer survivors to exercise programmes [36]. These barriers include a scarcity of available exercise programmes to refer to, as well as accessibility challenges related to cost and transport. The development of live-remote exercise programmes presents an opportunity to address some of these referral obstacles by expanding the availability of exercise programmes that healthcare professionals can refer cancer survivors to. Furthermore, future efforts should focus on enhancing training opportunities for healthcare professionals concerning exercise in cancer care.

The findings of the present study contribute to the understanding of the factors influencing exercise engagement among individuals treated for cancer, as well as how a live-remote exercise intervention can address certain barriers to engagement while leveraging facilitators. By mapping the identified themes onto the COM-B model, our study highlighted the interconnected roles of capability, opportunity, and motivation in shaping exercise engagement among participants. This study provides valuable insights that can help inform the future development and implementation of live-remote exercise interventions. Future efforts should consider the specific subtleties of live-remote exercise delivery, including allocating time and resources to ensure participants’ digital competency. Additionally, to provide psychosocial support in a virtual environment, a smaller group format with consistent participation throughout the intervention may be beneficial. Moreover, exercise professionals, who play a key role in creating this supportive environment, may need to employ alternative strategies suited to a virtual setting. Finally, potential privacy concerns in the home environment setup should also be addressed. Such interventions, while perhaps not being suitable for every individual treated for cancer, have the potential to significantly enhance the accessibility of exercise programmes for this population. Furthermore, these findings may be transferable to other digital health interventions designed for cancer survivors and be adaptable for use in interventions for individuals with other diseases.

Moving forward, it is recommended that future studies explore the implementation of live-remote exercise interventions as an alternative option alongside existing in-person exercise programmes. This approach could expand access to exercise programmes for individuals treated for cancer, thereby enabling broader participation and engagement in physical activity and exercise within this population.

## Limitations

The present study is subject to several potential methodological limitations that warrant consideration. Firstly, the participants were drawn from the intervention arm of an exercise trial conducted in Sweden, which may limit the generalisability of the findings and limit the reliability of cross-country comparisons. While the results offer a comprehensive understanding of the participant’s experiences, it is essential to acknowledge that this perspective may not fully represent the viewpoints of all individuals treated for cancer regarding factors influencing exercise engagement. Secondly, despite efforts to include females and males treated for breast, prostate, or colorectal cancer, our sample was predominantly composed of females with breast cancer (72.7%), resulting in an underrepresentation of males and those with prostate and colorectal cancer diagnoses. Thirdly, the average time between intervention completion and the focus group discussion was 19 weeks (SD 13.3). While none of the participants reported difficulty recalling information, this must be recognised as a potential limitation. Additionally, no formal member checking was conducted following data collection or analysis. Finally, internet access is widespread in Sweden, and digital health service utilisation is high, even among older individuals [37]. As such, caution should be taken when generalising the findings, as they may be influenced by the specific context, population characteristics, and time period of the study. Despite these limitations, it remains important to explore the exercise experiences of individuals treated for cancer to further develop interventions aimed at improving access to exercise programmes for individuals across different cancer types.

## Conclusion

The present study sheds light on exercise experiences post-cancer treatment, within the context of participating in a live-remote exercise intervention. It identifies factors influencing exercise engagement, highlighting the potential benefit of the live-remote format in overcoming certain barriers and fostering participation. Supervised by exercise professionals in group settings, live-remote exercise offers an alternative programme delivery method. The findings emphasise how the live-remote format provided participants with both psychosocial and exercise support, facilitating easier integration of exercise into their lives. Given the benefits of exercise in people treated for cancer, exploring alternative programme delivery methods is crucial for improving accessibility. The insights from the present study can inform the future development and implementation of live-remote exercise interventions for individuals with cancer.

## Supplementary information

### Supplementary Information

Below is the link to the electronic supplementary material.

Supplementary file1 (PDF 123 KB)

## Data Availability

The datasets used during the current study are available from the corresponding author on reasonable request.

## Abbreviations

HRQoL: Health-related quality of life
EX-MED Cancer: Exercise medicine programme for people with cancer
COM-B: Capability, opportunity, motivation model of behaviour

## References

[1] Sweegers MG, Altenburg TM, Brug J, May AM, van Vulpen JK, Aaronson NK et al (2019) Effects and moderators of exercise on muscle strength, muscle function and aerobic fitness in patients with cancer: a meta-analysis of individual patient data. Br J Sports Med 53(13):812. 10.1136/bjsports-2018-099191 30181323 10.1136/bjsports-2018-099191

[2] Stout NL, Baima J, Swisher AK, Winters-Stone KM, Welsh J (2017) A systematic review of exercise systematic reviews in the cancer literature (2005-2017). PM&R 9(9S2):S347–S384. 10.1016/j.pmrj.2017.07.074 28942909 10.1016/j.pmrj.2017.07.074 PMC5679711

[3] Mishra SI, Scherer RW, Geigle PM et al (2012) Exercise interventions on health-related quality of life for cancer survivors. Cochrane Database Syst Rev (8):CD007566. 10.1002/14651858.CD007566.pub2 22895961 10.1002/14651858.CD007566.pub2 PMC7387117

[4] McTiernan A, Friedenreich CM, Katzmarzyk PT, Powell KE, Macko R, Buchner D et al (2019) Physical activity in cancer prevention and survival: a systematic review. Med Sci Sports Exerc 51(6):1252–1261. 10.1249/mss.0000000000001937 31095082 10.1249/mss.0000000000001937 PMC6527123

[5] Friedenreich CM, Neilson HK, Farris MS, Courneya KS (2016) Physical activity and cancer outcomes: a precision medicine approach. Clin Cancer Res 22(19):4766–4775. 10.1158/1078-0432.Ccr-16-0067 27407093 10.1158/1078-0432.Ccr-16-0067

[6] Van Vulpen J, Sweegers MG, Peeters PHM, Courneya KS, Newton RU, Aaronson NK et al (2020) Moderators of exercise effects on cancer-related fatigue: a meta-analysis of individual patient data. Med Sci Sports Exerc 52(2):303–314. 10.1249/mss.0000000000002154 31524827 10.1249/mss.0000000000002154 PMC6962544

[7] Campbell KL, Winters-Stone KM, Wiskemann J, May AM, Schwartz AL, Courneya KS et al (2019) Exercise guidelines for cancer survivors: consensus statement from international multidisciplinary roundtable. Med Sci Sports Exerc 51(11):2375–2390. 10.1249/mss.0000000000002116 31626055 10.1249/mss.0000000000002116 PMC8576825

[8] Troeschel AN, Leach CR, Shuval K, Stein KD, Patel AV (2018) Physical activity in cancer survivors during “re-entry” following cancer treatment. Prev Chronic Dis 15:E65. 10.5888/pcd15.170277 29806579 10.5888/pcd15.170277 PMC5985854

[9] Thraen-Borowski KM, Gennuso KP, Cadmus-Bertram L (2017) Accelerometer-derived physical activity and sedentary time by cancer type in the United States. PLoS ONE 12(8):e0182554. 10.1371/journal.pone.0182554 28806753 10.1371/journal.pone.0182554 PMC5555687

[10] IJsbrandy, Hermens, Boerboom, Gerritsen, van Harten, Ottevanger, CRPMGLWMWRWHPB (2019) Implementing physical activity programs for patients with cancer in current practice: patients’ experienced barriers and facilitators. J Cancer Surviv 13(5):703–12. 10.1007/s11764-019-00789-3 31347009 10.1007/s11764-019-00789-3 PMC6828940

[11] Elshahat S, Treanor C, Donnelly M (2021) Factors influencing physical activity participation among people living with or beyond cancer: a systematic scoping review. Int J Behav Nutr Phys Act 18(1):50. 10.1186/s12966-021-01116-9 33823832 10.1186/s12966-021-01116-9 PMC8025326

[12] Clifford BK, Mizrahi D, Sandler CX, Barry BK, Simar D, Wakefield CE et al (2018) Barriers and facilitators of exercise experienced by cancer survivors: a mixed methods systematic review. Support Care Cancer 26(3):685–700. 10.1007/s00520-017-3964-5 29185105 10.1007/s00520-017-3964-5

[13] Gonzalo-Encabo P, Wilson RL, Kang DW, Normann AJ, Dieli-Conwright CM (2022) Exercise oncology during and beyond the COVID-19 pandemic: are virtually supervised exercise interventions a sustainable alternative? Crit Rev Oncol Hematol 174:103699. 10.1016/j.critrevonc.2022.103699 35526668 10.1016/j.critrevonc.2022.103699 PMC9069989

[14] Kotte M, Bolam KA, Mijwel S, Altena R, Cormie P, Wengström Y (2023) Distance-based delivery of exercise for people treated for breast, prostate or colorectal cancer: a study protocol for a randomised controlled trial of EX-MED Cancer Sweden. Trials 24(1):116. 10.1186/s13063-023-07152-z 36800978 10.1186/s13063-023-07152-z PMC9936694

[15] Michie S, van Stralen MM, West R (2011) The behaviour change wheel: a new method for characterising and designing behaviour change interventions. Implement Sci 6(1):42. 10.1186/1748-5908-6-42 21513547 10.1186/1748-5908-6-42 PMC3096582

[16] O’Brien BC, Harris IB, Beckman TJ, Reed DA, Cook DA (2014) Standards for reporting qualitative research: a synthesis of recommendations. Acad Med. 89(9):1245–1251. 10.1097/ACM.0000000000000388 24979285 10.1097/ACM.0000000000000388

[17] Braun V, Clarke V (2021) To saturate or not to saturate? Questioning data saturation as a useful concept for thematic analysis and sample-size rationales. Qualitative Research in Sport, Exercise and Health 13(2):201–216. 10.1080/2159676X.2019.1704846 10.1080/2159676X.2019.1704846

[18] Saarijärvi M, Bratt E-L (2021) When face-to-face interviews are not possible: tips and tricks for video, telephone, online chat, and email interviews in qualitative research. Eur J Cardiovasc Nurs 20(4):392–396. 10.1093/eurjcn/zvab038 33893797 10.1093/eurjcn/zvab038 PMC8135391

[19] Braun V, Clarke V (2021) Thematic analysis: a practical guide. Sage, London

[20] Braun V, Clarke V (2006) Using thematic analysis in psychology. Qual Res Psychol 3(2):77–101. 10.1191/1478088706qp063oa 10.1191/1478088706qp063oa

[21] Nowell LS, Norris JM, White DE, Moules NJ (2017) Thematic analysis: striving to meet the trustworthiness criteria. Int J Qual Methods 16(1):1609406917733847. 10.1177/1609406917733847 10.1177/1609406917733847

[22] Hansen AME, Hansen TF, Steffensen KD, Jensen LH (2020) Geographical distance as an impeding factor for cancer patients’ participation in a specialised exercise programme. Dan Med J 67(12):A01200044 33269696

[23] Haynes A, Gilchrist H, Oliveira JS, Sherrington C, Tiedemann A (2022) “I wouldn’t have joined if it wasn’t online”: understanding older people’s engagement with teleyoga classes for fall prevention. BMC Complement Med Ther 22(1):283. 10.1186/s12906-022-03756-1 36324148 10.1186/s12906-022-03756-1 PMC9628174

[24] Buffart LM, Kalter J, Sweegers MG, Courneya KS, Newton RU, Aaronson NK et al (2017) Effects and moderators of exercise on quality of life and physical function in patients with cancer: an individual patient data meta-analysis of 34 RCTs. Cancer Treat Rev 52:91–104. 10.1016/j.ctrv.2016.11.010 28006694 10.1016/j.ctrv.2016.11.010

[25] Fox L, Wiseman T, Cahill D, Beyer K, Peat N, Rammant E et al (2019) Barriers and facilitators to physical activity in men with prostate cancer: a qualitative and quantitative systematic review. Psycho-Oncology 28(12):2270–85. 10.1002/pon.5240 31617635 10.1002/pon.5240

[26] Rogers LQ, Vicari S, Courneya KS (2010) Lessons learned in the trenches: facilitating exercise adherence among breast cancer survivors in a group setting. Cancer Nursing 33(6):E10–E17. 10.1097/NCC.0b013e3181db699d 20562618 10.1097/NCC.0b013e3181db699d PMC2943976

[27] Craig BP, McDonough MH, Culos-Reed SN, Bridel W (2023) social support behaviours and barriers in group online exercise classes for adults living with and beyond cancer: a qualitative study. Curr Oncol 30(4):3735–3754. 10.3390/curroncol30040284 37185397 10.3390/curroncol30040284 PMC10136529

[28] Gui F, Tsai C-H, Vajda A, Carroll JM (2022) Workout connections: investigating social interactions in online group exercise classes. Int J Hum Comput Stud 166. 10.1016/j.ijhcs.2022.102870 10.1016/j.ijhcs.2022.102870

[29] Bland KA, Bigaran A, Campbell KL, Trevaskis M, Zopf EM (2020) Exercising in isolation? The role of telehealth in exercise oncology during the COVID-19 pandemic and beyond. Phys Ther 100(10):1713–1716. 10.1093/ptj/pzaa141 32737965 10.1093/ptj/pzaa141 PMC7454921

[30] Suderman K, Skene T, Sellar C, Dolgoy N, Pituskin E, Joy AA et al (2022) virtual or in-person: a mixed methods survey to determine exercise programming preferences during COVID-19. Curr Oncol 29(10):6735–6748. 10.3390/curroncol29100529 36290806 10.3390/curroncol29100529 PMC9601145

[31] Marthick M, McGregor D, Alison J, Cheema B, Dhillon H, Shaw T (2021) Supportive care interventions for people with cancer assisted by digital technology: systematic review. J Med Internet Res 23(10):e24722. 10.2196/24722 34714246 10.2196/24722 PMC8590193

[32] Chan RJ, Crichton M, Crawford-Williams F, Agbejule OA, Yu K, Hart NH et al (2021) The efficacy, challenges, and facilitators of telemedicine in post-treatment cancer survivorship care: an overview of systematic reviews. Ann Oncol 32(12):1552–1570. 10.1016/j.annonc.2021.09.001 34509615 10.1016/j.annonc.2021.09.001

[33] Brown M, O’Connor D, Murphy C, McClean M, McMeekin A, Prue G (2021) Impact of COVID-19 on an established physical activity and behaviour change support programme for cancer survivors: an exploratory survey of the Macmillan Move More service for Northern Ireland. Support Care Cancer 29(10):6135–6143. 10.1007/s00520-021-06165-1 33811517 10.1007/s00520-021-06165-1 PMC8019085

[34] Boot WR, Roque N, Charness NH, Rogers WA, Mitzner TL, Czaja SJ et al (2017) Older adult technology proficiency and technology adoption. Innov Aging 1(suppl_1):1026. 10.1093/geroni/igx004.3734 10.1093/geroni/igx004.3734

[35] Cantwell M, Walsh D, Furlong B, Loughney L, McCaffrey N, Moyna N et al (2020) Physical activity across the cancer journey: experiences and recommendations from people living with and beyond cancer. Phys Ther 100(3):575–585. 10.1093/ptj/pzz136 31588506 10.1093/ptj/pzz136

[36] Cantwell M, Walsh D, Furlong B, Moyna N, McCaffrey N, Boran L et al (2018) Healthcare professionals’ knowledge and practice of physical activity promotion in cancer care: challenges and solutions. Eur J Cancer Care 27(2):e12795. 10.1111/ecc.12795 10.1111/ecc.12795 29193416

[37] Internetstiftelsen. Svenskarna och internet 2022. Internetstiftelsen. 2022 https://svenskarnaochinternet.se/app/uploads/2022/10/internetstiftelsen-svenskarna-och-internet-2022.pdf. Accessed 25 Sep 2023.

