# Supplementary material for: The experience of live-remote exercise—perspectives after cancer treatment
Source: Support Care Cancer. 2024 Jul 18;32(8):526. doi: 10.1007/s00520-024-08736-4 (PMC11258172; doi:10.1007/s00520-024-08736-4)
Supplement: Supplementary file 1 — Supplementary file1 (PDF 123 KB) [file 520_2024_8736_MOESM1_ESM.pdf]

Supplement 1.

Main part of the interview guide containing questions on the experience of exercise among people treated for cancer who participated in the live-remote intervention of the EX-MED Cancer Sweden trial.

Key question	Probes
1. Tell us what made you decide to participate in a study about exercise?	• Motivation to exercise<br>• Anything that would make exercise more appealing<br>• Concerns exercising after a cancer diagnosis
2. What previous experience did you have with exercising (before joining the study)?	• Prior knowledge about exercising<br>• Impact of a cancer diagnosis and treatment<br>• Health concerns
3. What has it been like to exercise together in a group?	• Remotely compared to in-person<br>• With other cancer survivors, different diagnoses<br>• Mixed groups of men and women<br>• Group size<br>• Social aspects
4. How have the opportunities to engage in exercise been for you? For example, if you think about your work and family situation.	• Barriers to exercise - physical, mental, social<br>• Exercising in live-remote format<br>• Convenience, times<br>• Need for additional resources (e.g. computer/tablet, exercise equipment)
5. What advice, if any, have you previously received on physical activity and/or exercise?	• Guidance from health professionals<br>• Other sources of information
6. Please tell us about any effects of exercise that you experienced.	• Positive, negative<br>• Expectations prior
7. Is there anything else we have not discussed that is important for us to know?	
